# Acute effects of air pollution on all-cause mortality: a natural experiment from haze control measures in Chiang Mai Province, Thailand

**DOI:** 10.7717/peerj.9207

**Published:** 2020-05-27

**Authors:** Nitaya Vajanapoom, Patcharee Kooncumchoo, Thuan-Quoc Thach

**Affiliations:** 1 Center of Excellence in Global Health, Faculty of Public Health, Thammasat University, Pathumtani, Thailand; 2 Faculty of Allied Health Sciences, Thammasat University, Pathumtani, Thailand; 3 School of Public Health, The University of Hong Kong, Hong Kong, China

**Keywords:** Haze smoke, Biomass burning, Air pollution, Particulate matter, Mortality, Health effects, Haze control

## Abstract

**Background:**

Serious haze episodes have been a seasonal event in Chiang Mai province for more than a decade. In 2008, local government agencies introduced comprehensive measures to control haze and limit its impacts on public health. This study assessed the acute effects of ambient air pollutants on all-cause mortality before and after the introduction of those haze control measures.

**Methods:**

We obtained daily mortality counts and data on mass concentrations of particulate matter <10 micron in aerodynamic diameter (PM_10_), gaseous pollutants (SO_2_, NO_2_, O_3_, and CO), and meteorology in Chiang Mai Province between January 2002 and December 2016. We analyzed the data using a case-crossover approach adjusting for temperature, relative humidity, seasonality, and day-of-week. We assessed change in the excess risks of all-cause mortality associated with an increase in interquartile range (IQR) of pollutant concentration before and after control measures came into force.

**Results:**

We found decreased PM_10_ levels and markedly reduced excess risks of daily mortality associated with an IQR increase in PM_10_ concentrations in the years after haze-control measures were implemented (2009–2016). We found mixed results for gaseous pollutants: SO_2_ showed no significant change in excess risk of daily mortality throughout the study period, while NO_2_ and CO showed significant excess risks only in the period 2012–2016, and 8-h maximum O_3_ showed a decrease in excess risk despite an increase in its atmospheric levels after the introduction of haze control measures in 2008.

**Conclusions:**

The findings indicate that the government haze control measures first introduced in Chiang Mai province in 2008 have successfully reduced episodic PM_10_ concentrations, which has led to a decrease in short-term all-cause mortality.

## Introduction

Biomass burning is a significant source of haze smoke worldwide. Haze smoke comprises many agents that can be harmful to public health, such as particulate matter (PM), nitrogen dioxide (NO_2_), ozone (O_3_), carbon monoxide (CO), polycyclic aromatic hydrocarbons (PAHs), and volatile organic compounds (VOCs) (Chen et al., 2017; Naeher et al., 2007). Smoke plumes from biomass burning can raise ambient particulate matter levels many times higher than that observed on normal days (Liu et al., 2015).

Recent systematic reviews have consistently reported associations of smoke plume-related PM with both respiratory morbidity and all-cause mortality (Cascio, 2018; Liu et al., 2015; Reid Colleen et al., 2016). Other studies have examined the effects of transboundary smoke plume-related PM_2.5_ from the 2002 Quebec wildfires in Canada to the east coast of US (Le et al., 2014; Zu et al., 2016), and in eastern countries of Europe to Helsinki, Finland (Kollanus et al., 2016). These studies found no effects of transboundary smoke plume relalted-PM_2.5_ on mortality (Kollanus et al., 2016; Zu et al., 2016), but impacts on respiratory and cardiovascular hospitalization rates were documented (Le et al., 2014). In addition, the 1997 South East Asia forest fires (mainly from Indonesia) caused an increased incidence of respiratory illnesses in Singapore (Emmanuel, 2000; Mott et al., 2005).

Despite an extensive literature addressing the health effects of haze smoke episodes, the health impacts of haze smoke-related gaseous pollutants are rarely reported. One example is a study from Portugal which recorded levels of CO, NO_2_, VOCs in the exhaled breath of firefighters during firefighting activities. Medical tests showed that the increase in CO and decrease in NO in exhaled air found in the majority of the firefighters were beyond the limits recommended by Occupational Exposure Standard values (Miranda et al., 2012).

Haze episodes have been recognized as a seasonal event in northern Thailand for more than a decade, caused mainly by agricultural burning and forest fires. Open burning is commonly practiced during the hot season (February–April) when villagers begin preparing their fields for the next crop. Government attention to the problem first began after the haze episode of 2007. This event was particularly severe, emitting significant amounts of haze smoke in several provinces of northern Thailand, during which PM_10_ levels in Chiang Mai province exceeded the WHO interim target-1 standard (150 µg/m^3^ 24-hour) by several times.

Following this event, comprehensive haze-control measures were implemented by government agencies in all northern provinces of Thailand, including Chiang Mai province. These measures comprised a series of prevention and control actions with the primary aim of reducing haze smoke emissions, which included conducting agriculture burning before the peak burning period, enforcing a ban on biomass burning during the peak burning period, reducing forest wastes to prevent forest fires, and monitoring hot spots to gain early control over agriculture burning and forest fires.

In Chiang Mai province, these measures have been fully implemented since early 2008. The Province offers a unique opportunity to conduct a natural experimental study to investigate the potential impacts of the implementation on health risk of a well-defined intervention to reduce air pollution. Thus, the purpose of this study is to assess the short-term association between selected criteria air pollutants and daily mortality, focusing on the changes of the excess risk of daily mortality associated with these air pollutants, over the period 2002 to 2016.

## Materials & Methods

### Study area and period

We assessed the impacts of governmental haze control measures on the acute effects of air pollution and daily mortality in Chiang Mai province, which is located in northern Thailand and is surrounded by high mountain ranges. Chiang Mai province covers an area of 20,170 km^2^ and has the population growth from 1.60 million in 2002 to 1.74 million in 2016 (Thailand Department of Provincial Administration, 2020). The period examined by this study is from 2002 to 2016.

### Mortality data

We obtained daily mortality data for Chiang Mai province from 1 January 2002 to 31 December 2016 from the Ministry of Public Health. We included all-cause mortality in the study. [International Classification of Diseases, 10th Revision ICD-10 due to all-natural causes (A00-R99)]. Methods for data collection and quality control are described elsewhere (Vichit-Vadakan, Vajanapoom & Ostro, 2008).

### Air pollution and meteorologic data

We obtained data on hourly concentrations of particulate matter with aerodynamic diameter ≤10 µm (PM_10_), sulphur dioxide (SO_2_), nitrogen dioxide (NO_2_), ozone (O_3_), and carbon monoxide (CO) for the Period 1 January 2002 to 31 December 2016 from two fixed-site general monitoring stations operated by the Pollution Control Department, Ministry of Natural Resources and Environment. The two monitoring stations are located in Chang Peuag sub-district (UTM 18.840732, 98.969780) and Sri Meaung sub-district (UTM 18.7909333, 98.99), Chiang Mai province. The measurement methods used for PM_10_, SO_2_, NO_2_, O_3_, and CO were beta ray, ultraviolet fluorescence, chemi-luminescence, ultraviolet absorption photometry, and non-dispersive infrared detection, respectively. We averaged the hourly concentrations of air pollutants from the two monitoring stations to represent hourly concentrations of the entire area of Chiang Mai province. We computed daily concentrations of air pollutants by calculating the 24-h average for PM_10_, NO_2_, SO_2_, CO, and moving 8-h maximum O_3_. In computing hourly and daily data, at least 6 out of 8 h data were required for moving 8-h maximum O_3_ and 18 h or more for PM_10_, SO_2_, NO_2_, and CO.

We obtained data on daily mean temperature and relative humidity from three monitoring stations across Chiang Mai province from the Meteorological Department of the Ministry of Digital Economy and Society. We averaged the 24-h mean temperature and relative humidity from the three monitoring stations to determine daily temperature and relative humidity for Chiang Mai province.

### Statistical method

The statistical analysis method was previously described in Thach et al. (2018). In brief, we evaluated short-term the relationship between the daily air pollutants and non-accidental mortality using a time-stratified case-crossover approach, in which the day of each death is considered as the case and all other days within the same calendar month as controls. We used conditional logistic regression to assess associations between mortality and air pollutants adjusted for mean relative humidity, mean temperature up to lag 14 days, seasonality, and day of week. We included mean temperature in the regression using a natural spline of 3 degrees of freedom and mean relative humidity was entered into the model as linear effects. To maximize the power of the study, the effect of day of the week was controlled for using indicator variables in the model, in place of the approach of matching (Janes, Sheppard & Lumley, 2005; Schwartz, 2004). To help interpretation, we transformed the odds ratios to excess risks defined as the percentage change in mortality associated with an increase in interquartile range pollutant concentration and can be approximated by (odds ratio − 1) × 100% (Janes, Sheppard & Lumley, 2005).

We calculated exposure lags up to 3 days for the air pollution data. In addition, we calculated the means of lags 0–1 day for the air pollution data.

We divided the 15-year study period into 3 sub-periods: Period 1 from 1 January 2002 to 31 December 2007; period 2 from 1 January 2008 to 31 December 2011, and Period 3 from 1 January 2012 to 31 December 2016. period 1 covered the time before haze control measures were in force. periods 2 and 3 were both after haze control measures were introduced, where Period 2 was the time during the 2008–2011 action plan of the governmental haze control measures were implemented (Department of Pollution Control, 2010), and Period 3 was the time from 2102 to 2016. We analyzed the data separately for each period.

To explore the robustness of the model, we carried out a sensitivity analysis allowing for effects of day of week by matching, rather than explicit control for, in the model. All statistical analyses were conducted using the statistical software R version 3.3.1 (R Core Team, 2016).

## Results

**Figure 1:**
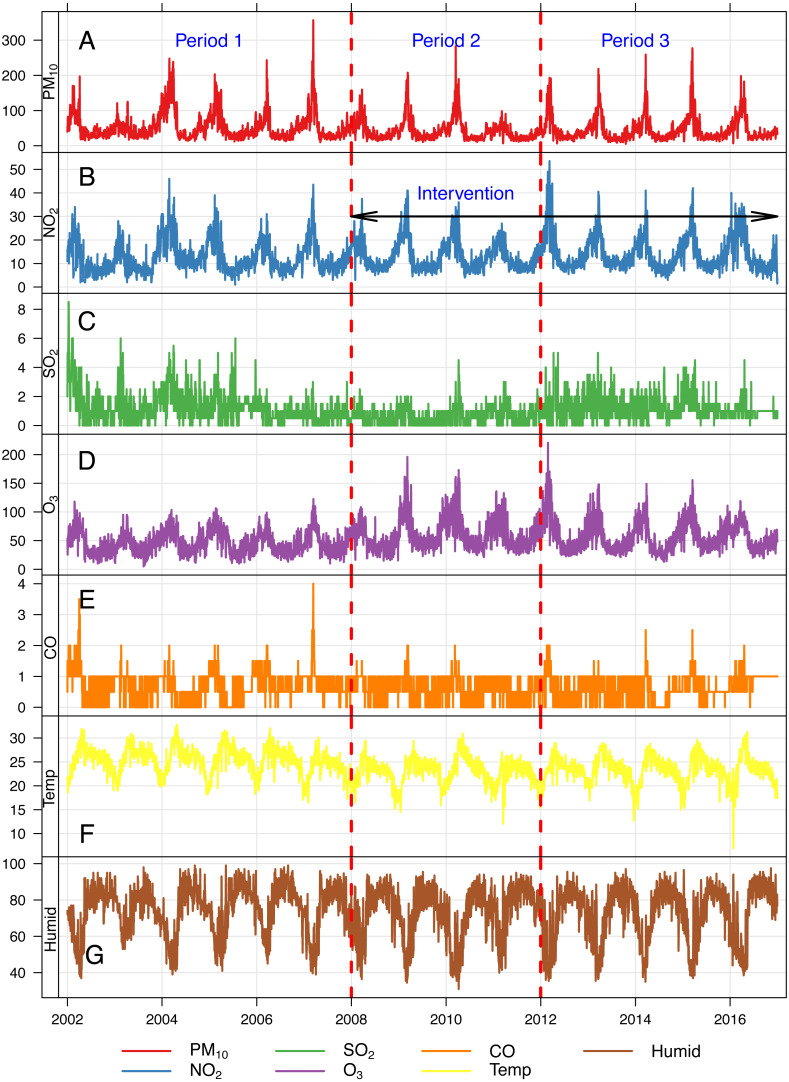
Daily concentrations of air pollutants and meteorological data in Chiang Mai province, Thailand, between 2002–2016. (A) PM_10_, (B) NO_2_, (C) SO_2_, (D) O_3_, (E) Temp, (F) Humid.Period 1, 1/1/2002–31/12/2007. Period 2, 1/1/2008–31/12/2011. Period 3, 1/1/2012–31/12/2016. Temp, daily mean temperature. Humid, daily mean relative humidity (%).

Over the 15-year study period, an average of about 35 deaths per day from all-causes was observed in Chiang Mai Province (Table 1). We found a reduction in mean concentrations of PM_10_ during the haze episodes period of February to April. The evidence is more prominent in the early period of the haze control intervention (from 2008 to 2011, especially in 2011) than the later period (from 2012 to 2016) (Fig. 1).

**Table 1:** Descriptive statistics of all-cause daily death counts in Chiang Mai province, between 2002 and 2016.

Period	Days	Mean (sd)
1	2,191	35.1 (7.4)
2	1,461	33.5 (6.7)
3	1,826	34.8 (7.1)

**Notes.**

Period 1: 1/1/2002–31/12/2007. Period 2: 1/1/2008–31/12/2011. Period 3: 1/1/2012–31/12/2016.

All pollutants showed seasonal variations, with higher concentrations in the summer season and lower concentrations in the rainy season. On some days, daily mean PM_10_ concentrations were higher than the Interim Target 1 (IT-1) of WHO air quality guideline (not exceeding 150 µg/m^3^) (Fig. 1). Table 2 shows the mean differences between individual pollutant concentrations. Generally, we found higher concentrations of PM_10_, SO_2_, and CO before the introduction of control measures, and an immediate and sustained decrease after their introduction (2008–2016). The decrease was greater in Period 2 than in Period 3, especially for PM_10_ in which a mean difference of 12.18 µg/m^3^ (95% CI [9.94–14.41]) and 9.11 µg/m^3^ (95% CI [6.86–11.36]) was found for period 2 and period 3 respectively. In contrast, the concentrations of 8-h maximum O_3_ and NO_2_ were lower before controls were introduced, and slightly higher afterwards.

Table 3 presents the regression results of the estimated excess risks of all-cause mortality for the 3 periods. We found that the highest excess risk of 3.01% (95% CI [1.49–4.55]) of all-cause mortality for one IQR increase in lag 0-1 PM_10_ was in Period 1. We further found a substantial decrease of the excess risk in period 2. Excess risks of lag2 PM_10_ = 0.81% (95% CI [−0.97–2.62]) and lag3 PM_10_ = 1.45% (95% CI [−0.03–2.94]) were seen in Period 2 and Period 3 respectively; but none of them was statistically significant.

The changes in the short-term effects of gaseous pollutants on all-cause mortality are mixed. We found no significant changes in the excess risks for SO_2_ over the 3 study periods, while several lags of NO_2_ and lag2 CO exhibited an increased excess risk only in Period 3. On the contrary, larger excess risks for several lags of 8-h maximum O_3_ were seen during the Period 1, and these subsequently decreased after 2008.

**Table 2:** Descriptive statistics of air pollutants in Chiang Mai province, between 2002 and 2016.

**Air pollutants**	**Period**	**Days**	**Missing days**	**Mean(95% CI)**	Mean Δ_(1−2)_**(95% CI)**	Mean Δ_(1−3)_**(95% CI)**
PM_10_ (µg/m^3^)	1	2,165	26	54.34 (52.67, 56.01)	12.18 (9.94, 14.41)^*^	0.11 (6.86, 11.36)^*^
	2	1,461	0	42.16 (40.61, 43.71)		
	3	1,823	4	45.23 (43.66, 46.80)		
SO_2_ (ppb)	1	2,181	10	1.27 (1.23, 1.31)	0.60 (0.55, 0.65)^*^	0.07 (0.01, 0.12)^*^
	2	1,460	1	0.68 (0.65, 0.71)		
	3	1,797	30	1.21 (1.18, 1.24)		
NO_2_ (ppb)	1	2,164	27	12.41 (12.13, 12.69)	−0.63 (−1.05, −0.21)^*^	−1.87 (−2.30, −1.44)^*^
	2	1,461	0	13.04 (12.72, 13.36)		
	3	1,821	6	14.28 (13.94, 14.62)		
8-h max O_3_ (ppb)	1	2,170	21	34.77 (34.11, 35.43)	−6.98 (−8.09, −5.87)^*^	−6.17 (−7.27, −5.08)^*^
	2	1,457	4	41.76 (40.84, 42.68)		
	3	1,813	14	40.95 (40.05, 41.85)		
CO (ppm)	1	2,181	10	0.71 (0.69, 0.73)	0.11 (0.09, 0.13)^*^	0.05 (0.03, 0.07)^*^
	2	1,459	2	0.60 (0.59, 0.61)		
	3	1,819	8	0.65 (0.64, 0.66)		

**Notes.**

* Statistically significant for independent *t*-test, *p* value < 0.05.

Δ_(1−2)_ Period 1–Period 2 Δ_(1−3)_ Period 1–Period 3

Period 1: 1/1/2002–31/12/2007. Period 2: 1/12008–31/12/2011. Period 3: 1/1/2012–31/12/2016.

**Table 3:** Percent excess risk (ER) of all-cause mortality for one IQR change in air pollutant levels.

**Pollutants**	**IQR**	**Period 1Before intervention**		**Period 2After intervention**		**Period 3After intervention**	
		**% ER**	**95% CI**	**% ER**	**95% CI**	**% ER**	**95% CI**
**PM**_10_**(µg/m^3^)**							
Lag 0	33.0	2.19	0.83, 3.57	−0.60	−2.53, 1.36	0.58	−0.99, 2.17
Lag 1	33.0	1.19	−0.15, 2.54	0.63	−1.20, 2.49	0.76	−0.74, 2.29
Lag 2	33.0	0.79	−0.54, 2.14	0.81	−0.97, 2.62	1.23	−0.26, 2.74
Lag 3	33.0	0.09	−1.20, 1.41	−0.10	−1.85, 1.68	1.45	−0.03, 2.94
Lag 0–1	32.3	3.01	1.49, 4.55	0.06	−1.92, 2.08	0.61	−1.05, 2.29
**NO**_2_**(ppb)**							
Lag 0	8.0	−0.60	−2.38, 1.20	−0.67	−3.08, 1.79	1.63	−0.39, 3.69
Lag 1	8.0	−0.20	−1.96, 1.58	−0.69	−2.97, 1.65	1.72	−0.28, 3.76
Lag 2	8.0	−0.70	−2.42, 1.05	1.53	−0.72, 3.83	2.44	0.46, 4.45
Lag 3	8.0	−1.31	−2.99, 0.39	1.41	−0.82, 3.69	1.72	−0.24, 3.72
Lag 0–1	8.0	1.59	−0.57, 3.79	−0.84	−3.43, 1.83	2.38	0.07, 4.74
**SO**_2_**(ppb)**							
Lag 0	1.0	−0.47	−1.52, 0.60	−0.87	−2.79, 1.10	−0.60	−1.89, 0.71
Lag 1	1.0	−0.64	−1.69, 0.42	−0.77	−2.70, 1.20	−0.09	−1.39, 1.23
Lag 2	1.0	−0.13	−1.18, 0.94	−1.23	−3.14, 0.71	−0.80	−2.08, 0.49
Lag 3	1.0	0.77	−0.30, 1.85	1.16	−0.79, 3.16	−0.25	−1.51, 1.02
Lag 0–1	1.0	0.08	−1.17, 1.34	−1.62	−3.99, 0.82	−1.00	−2.71, 0.74
**8-h max O**_3_**(ppb)**							
Lag 0	31.0	3.55	1.28, 5.88	−0.47	−2.21, 1.30	−0.07	−1.99, 1.89
Lag 1	31.0	3.12	0.91, 5.38	−0.47	−2.21, 1.30	1.74	−0.18, 3.71
Lag 2	31.0	2.04	−0.12, 4.24	0.55	−1.14, 2.27	3.04	1.12, 4.99
Lag 3	31.0	−0.26	−2.35, 1.87	−0.83	−2.98, 1.36	1.85	−0.03, 3.77
Lag 0–1	30.5	5.65	2.88, 8.49	−0.82	−2.93, 1.34	0.01	−2.27, 2.34
**CO (ppm)**							
Lag 0	0.5	−0.20	−1.46, 1.08	0.22	−1.33, 1.79	−0.20	−1.59, 1.22
Lag 1	0.5	−0.76	−2.00, 0.50	0.84	−0.72, 2.43	−0.03	−1.42, 1.38
Lag 2	0.5	−0.97	−2.20, 0.28	−0.47	−2.00, 1.08	1.61	0.20, 3.05
Lag 3	0.5	−0.85	−2.06, 0.39	0.02	−1.51, 1.58	0.86	−0.53, 2.28
Lag 0–1	0.5	0.01	−1.53, 1.58	0.80	−1.28, 2.93	0.43	−1.38, 2.28

**Notes.**

Period 1:1/1/2002–31/12/2007. Period 2: 1/1/2008–31/12/2011. Period 3: 1/1/2012–31/12/2016.

### Sensitivity analysis

We carried out a sensitivity analysis allowing for effects of day of the week by matching, rather than explicit control for, in the model. There were minimal changes in the excess risks, but the direction of the findings did not change (Table 4).

**Table 4:** Sensitivity analyses for all-cause mortality associated with one IQR change in air pollutants.

**Pollutants**	**IQR**	**Period 1Before intervention**		**Period 2After intervention**		**Period 3After intervention**	
		**% ER**	**95% CI**	**% ER**	**95% CI**	**% ER**	**95% CI**
**PM**_10_**(µg/m^3^)**							
Lag 0	33.0	2.26	0.90, 3.64	−0.55	−2.47, 1.41	0.64	−0.92, 2.23
Lag 1	33.0	1.01	1.00, 1.03	0.48	−1.33, 2.33	0.39	−1.10, 1.91
Lag 2	33.0	0.67	−0.65, 2.01	0.77	−1.00, 2.57	1.05	−0.43, 2.56
Lag 3	33.0	0.03	−1.27, 1.34	−0.05	−1.80, 1.73	1.42	−0.05, 2.91
Lag 0–1	32.3	2.97	1.46, 4.51	0.00	−1.97, 2.01	0.56	−1.09, 2.23
**NO**_2_**(ppb)**							
Lag 0	8.0	−0.71	−2.47, 1.09	−0.61	−2.99, 1.84	2.60	0.60, 4.63
Lag 1	8.0	−0.80	−2.53, 0.96	−1.00	−3.26, 1.31	0.29	−1.64, 2.27
Lag 2	8.0	−1.15	−2.85, 0.58	1.52	−0.69, 3.79	0.80	−1.11, 2.75
Lag 3	8.0	−1.21	−2.88, 0.48	1.52	−0.69, 3.79	0.51	−1.39, 2.46
Lag 0–1	8.0	1.86	−0.28, 4.06	−1.01	−3.59, 1.64	2.97	0.68, 5.30
**SO**_2_**(ppb)**							
Lag 0	1.0	−0.50	−1.56, 0.56	−0.85	−2.77, 1.11	−0.41	−1.70, 0.90
Lag 1	1.0	−0.58	−1.63, 0.48	−0.83	−2.75, 1.13	−0.24	−1.53, 1.08
Lag 2	1.0	0.03	−1.03, 1.10	−1.36	−3.26, 0.57	−0.93	−2.20, 0.35
Lag 3	1.0	0.76	−0.31, 1.84	1.22	−0.73, 3.20	−0.13	−1.38, 1.14
Lag 0–1	1.0	−0.04	−1.29, 1.22	−1.65	−4.02, 0.77	−0.74	−2.46, 1.00
**8-h max O**_3_**(ppb)**							
Lag 0	31.0	3.55	1.27, 5.87	−0.52	−2.25, 1.24	0.49	−1.42, 2.44
Lag 1	31.0	3.03	0.82, 5.28	−0.52	−2.25, 1.24	0.89	−1.00, 2.83
Lag 2	31.0	2.09	−0.06, 4.30	0.45	−1.23, 2.16	1.53	−0.36, 3.44
Lag 3	31.0	−0.21	−2.30, 1.92	−0.76	−2.91, 1.42	0.83	−1.02, 2.71
Lag 0–1	30.5	5.62	2.85, 8.46	−0.75	−2.86, 1.40	0.74	−1.53, 3.06
**CO (ppm)**							
Lag 0	0.5	−0.48	−1.73, 0.79	0.31	−1.22, 1.87	0.12	−1.27, 1.53
Lag 1	0.5	−1.00	−2.24, 0.25	0.58	−0.95, 2.14	−0.02	−1.40, 1.39
Lag 2	0.5	−0.99	−2.21, 0.26	−0.49	−2.00, 1.05	1.29	−0.12, 2.72
Lag 3	0.5	−0.54	−1.75, 0.70	0.05	−1.47, 1.59	0.54	−0.84, 1.95
Lag 0–1	0.5	−0.15	−1.69, 1.41	0.65	−1.40, 2.75	0.37	−1.44, 2.20

**Notes.**

Period 1: 1/1/2002–31/12/2007. Period 2: 1/1/2008–31/12/2011. Period 3: 1/1/2012–31/12/2016.

## Discussion

Agriculture burning and forest fires are the major cause of haze smoke and episodic PM_10_ pollution in Chiang Mai (Punsompong & Chantara, 2018; Tsai et al., 2013). Our analyses have shown that the governmental haze-control measures introduced in Chiang Mai in 2008 have resulted in a sustained reduction in episodic ambient PM_10_ levels. The reduction in PM_10_ levels observed in Period 2, (particularly pronounced during 2011) was greater than that observed in Period 3. We further found a substantial decrease of short-term association of mortality for an IQR increase in PM_10_ levels after 2008.

A most recent study demonstrated that substantial reductions in PM_2.5_ levels could have been achieved in northeast China in 2015, where biomass burning is a major emission source of PM_2.5_ during harvest season, if a ban on biomass burning had been implemented in the region that year (Yang et al., 2020). The reduction in PM_10_ levels after 2008 found in our results accorded with the evidence found by Yang et al. (2020), suggesting the beneficial impacts of the haze control meausres on reducing PM_10_ levels in Chiang Mai.

During the period of our study, there may have been other factors that contributed to the observed changes in PM_10_ levels. Climatological conditions may have contributed to the observed changes. In particular, the large reduction in PM_10_ concentrations observed in 2011 could be partly due to the unusually heavy monsoon rains and unprecedented levels of flooding across much of Thailand that year. In addition, long-range transport of smoke plumes from surrounding areas could explain the rising PM_10_ levels observed in all non-accidental 3. Forest fire events occurred throughout South and Southeast Asia during each dry season from 2001 to 2015 (Chen et al., 2017). It has been shown that there is heavy air movement from the south and southwest direction passing through many hotspot areas in Chiang Mai by the event on 21 March 2015 (Phairuang et al., 2019), and that PM_10_ is driven into the Chiang Mai Basin by southwest winds from Thai-Myanmar border (Kiatwattanacharoen et al., 2017).

The results also show that as the PM_10_ levels changed over time, the excess risks estimates also changed. The findings could be explained by a plausible change in PM_10_ composition as a result of the haze-control measures, and that is reflected in the change in the excess risk estimates. We cannot exclude chance from the findings, but this should be of less concern due to the long study period of our analysis.

During a 13 month strike at a local mill in Utah Valley in the USA, PM_10_ concentration in the area was reduced by 15 µg/m^3^ and total deaths were decreased by 3.2% (Pope, Schwartz & Ransom, 1992). If PM_10_ concentration there had dropped by 12.8 µg/m^3^ as was found in Chiang Mai during study Period 2 (Table 2), we would expect to see total mortality in Utah Valley reduced by 2.7%. Our results show a 2.1% reduction in excess mortality from all causes during period 2 (Table 3), approximately equal to the estimation for Utah Valley. The findings indicate a consistency of our results with a previous known natural experimental study investigating the impacts of air pollution interventions on all-cause mortality.

We found mixed results for gaseous pollutants. The results showed a slightly decreasing pattern of SO_2_ and CO levels over the study period, and they were not statistically associated with daily mortality over the study period. We found higher levels for NO_2_ and 8-h maximum O_3_ after 2008 than for the levels found in Period 1, and they were both statistically associated with daily mortality in Period 3. Thus, the impact of the haze-control measures on ambient gaseous pollutant levels and mortality are inconclusive, which may indicate that ambient gaseous pollutants in Chiang Mai are more likely emitted by non-haze related sources. It has been noted that NO_2_ and SO_2_ are always associated with vehicle emission and industrial emission (Fujita et al., 2003; Rai et al., 2011). Although there are many gaseous materials in smoke emissions, such as CO, NO_2_, O_3_ (Chen et al., 2017; Naeher et al., 2007), they are commonly changed into particles by gas-phase oxidation (Cheng et al., 2013; DeCarlo et al., 2010) during migration from smoke generating areas to surrounding areas. Further, either decreased or increased O_3_ levels was observed in fire-affected areas (Alvarado et al., 2010). Therefore, trace gases contained in haze smoke episodes may not be a significant contributor to ambient gaseous pollutant levels in Chiang Mai province.

To date, studies on the health impacts of wildfire-related trace gases are scarce. Moreover, they have been limited to the health effects of O_3_, and their results are inconclusive. Azevedo, Gonçalves & De Fátima Andrade (2011) documented that during the 2005 forest fires on the northern coast of Portugal, peaks in O_3_ concentration were positively associated with high rates of hospital admission from cardiovascular diseases, but not for respiratory diseases in the metropolitan area of Porto. Another study examined the impacts of bushfire smoke events in Sydney from 1997 to 2004 on mortality (Johnston et al., 2011). The authors did not find the change in the association between bushfire smoke events and non-accidental mortality after including 1-h maximum O_3_ in the regression model, indicating no independent effect of 1-h maximum O_3_ on mortality. Our results are broadly inconsistent with the results from this study, as we found the association between 8-h maximum O_3_ and daily mortality both in Period 1 and Period 3. Accordingly, no conclusive evidence can be drawn from the current data for the health impacts of O_3_ in an episodic haze smoke area.

Research has consistently demonstrated an association between change in urban air pollution levels and change in health outcomes, through implementation of air quality regulatory programs targeting sources of pollution. Examples of such programs are: replacing coal brown to natural gas in Erfurt, Germany (Breitner et al., 2009), banning bituminous coal in Dublin, Ireland (Clancy et al., 2002); more stringent national emission standards for vehicles in the USA (Dominici et al., 2007); and reduction in sulfur content in fuel in Hong Kong (Hedley et al., 2002). However, the impacts of long-term implementation of biomass burning emission control are rarely studied, especially in Southeast Asia where biomass burning is of great concern to environmental and public health (Liu et al., 2015). Our results have added to the literature new knowledge on the beneficial impacts of haze-control measures on air quality and mortality rates after a long period of the implementation.

Our regression results are robust as the sensitivity analysis results did not change significantly after maximizing the power of the study by using indicator variables for days of the week in the model, rather than the more common approach of matching (Janes, Sheppard & Lumley, 2005; Schwartz, 2004). However, there are a few limitations to our analysis. For instance, the air pollution data from the two-fixed air monitoring sites may not be representative of the entire area of Chiang Mai province, especially, for spatial heterogeneous pollutants like CO, NO_2_ (Sarnat et al., 2010). Hence, their risk estimates might be attenuated as a result of the possible non-differential error of population exposure. This potential measurement error is of less concern for O_3_ and PM_2.5_ as they were homogeneously dispersed in the ambient (Sarnat et al., 2010), and this could be the same for PM_10_ because its mass concentration consisted of a relatively high fraction (74%) of PM_2.5_ (Amnuaylojaroen & Kreasuwun, 2011). Moreover, even though Chiang Mai province may mainly be impacted by the biomass burning, the actual changes such as regional transferring air pollution should not be overlooked. However, we did not have data available to examine the influence of long-range travel haze smoke from surrounding provinces and neighboring countries on the ambient air pollution levels in Chiang Mai province. These external sources may reduce the effectiveness of the mitigation of biomass burning emission in the province.

## Conclusions

The findings indicate that the government haze control measures first introduced in Chiang Mai province in 2008 have successfully reduced episodic PM_10_ concentrations, which has in turn lead to a decrease in short-term all-cause mortality associated with high PM_10_ levels. Further, we observed that significant changes were seen immediately after introduction of the control measures. However, no conclusion was able to be drawn on the impacts of those measures on gaseous pollutants. Evaluating the effects of biomass control measures on cause-specific mortality is suggested for future researches.

##  Supplemental Information

10.7717/peerj.9207/supp-1 File S1 Acute effects of air pollution on all-cause mortality

## Additional Information and Declarations

### Data Availability

The following information was supplied regarding data availability: The raw data are available in the Supplemental Files.
